# An Industry-Related Outbreak of Human Anthrax: Massachusetts, 1868

**DOI:** 10.3201/eid0810.010509

**Published:** 2002-10

**Authors:** Abe Macher

**Affiliations:** Health Resources and Services Administration, U.S. Public Health Service, Rockville, Maryland, USA

**To the Editor:** In Bioterrorism-Related Inhalational Anthrax: The First 10 Cases Reported in the United States, Jernigan et al. noted that in the mid-1800s inhalational anthrax related to the textile industry became known as woolsorters’ disease (in England) and ragpickers’ disease (in Germany and Austria) because of the frequency of infection in mill workers exposed to imported animal fibers contaminated with *Bacillus anthracis* spores (1). During the 1800s, as in Europe, industry-related human cases of anthrax also occurred in the United States.

In 1868, Silas Stone, a physician, reported that “an unusual number of cases of a rather rare affection have come under my observation within the past 14 months” (2). Stone described eight patients with “malignant pustules” who worked in or were associated with an animal hair factory in Massachusetts. The patients’ cutaneous lesions were described as dark red, dark purple, purplish-black, and black; six of the patients had “slough” lesions. Stone treated his patients with tincture of iodine, iron, and quinine. Since antibiotics were not available, six of the eight patients had severe clinical disease, and two died. Stone’s patients demonstrated the full spectrum of anthrax, including gastrointestinal, mediastinal, and meningeal involvement. Four patients had gastrointestinal symptoms, including epigastric distress and pain, nausea, and vomiting. Three patients had mediastinal involvement, manifested by chest distress and pain, dyspnea, and tachypnea. In the two fatal cases, meningitis appeared to have been the immediate cause of death; both of these patients were described as delirious.

Among Stone’s eight patients, most remarkable was case 5, which was strikingly similar to case 8 of Jernigan et al.; the signs and symptoms of both patients included chills, headache, fatigue, vomiting, chest pain, tachypnea, tachycardia, and cutaneous lesions. Stone’s description of the 7-day clinical course of patient 5, a laborer at the hair factory, is as follows: “Called November 17. Had been sick since the Thursday previous (November 14). Was taken with chills, pain in head and back, and suffered loss of strength. When first seen, was in bed . . . had not slept well the previous night. Pain and distress in epigastrium and back. Pulse 120 . . . breathing hurried. Discovered a dark purple spot surrounded by yellow vesicles . . . pressure on slough produced no pain. November 18: Slough doubled in size. November 19: Vomited . . . severe chill. November 20: Sleep restless . . . slough one inch by half an inch, much raised above surrounding skin, with a red areola about an inch in width. November 21: a.m.: Delirious part of night . . . slept but little . . . pain in chest. 3 p.m.: Distress at epigastrium great . . . delirium more violent. 8 p.m.: Distress and delirium greater . . . pulse failing . . . sinking rapidly . . . died soon after visit.”

Stone perceptively noted that each of his patients was directly or indirectly exposed to hair or dirt from the animal hair factory, and that in the surrounding population not so exposed, no cases were seen. Stone realized that he was dealing with an industry-related disease and hypothesized that the cause was “a specific poison, and not simply putrescent animal matter.” Nine years after Stone’s 1868 report, Robert Koch in Germany reported isolation and cultivation of *B. anthracis*, the formation of its spores, the production of anthrax disease with pure cultures, and the recovery of *B. anthracis* from experimental infection (3).
